# Editorial

**DOI:** 10.1111/acel.12187

**Published:** 2014-01-16

**Authors:** Peter Adams, Adam Antebi, Ana Maria Cuervo, Brian Kennedy, John Sedivy

**Affiliations:** 1 Beatson InstituteGlasgow, UK; 2 Max Planck Institute for Biology of AgeingKoeln, Germany; 3 Albert Einstein College of MedicineBronx, NY, USA; 4 Buck Institute for Research on AgingNovato, CA, USA; 5 Brown UniversityProvidence, RI, USA

## Aging Cell: Open Access to all

Since its launch in 2002, *Aging Cell* has strived to publish the highest caliber research in biogerontology (now often referred to as the biology of aging). *Aging Cell* has rapidly established itself as a highly respected and reputable journal and has maintained its leadership role in publishing some of the most important and exciting research in this field.

As we enter 2014 (the thirteenth year of our publication), *Aging Cell* will undergo some changes that we wish to introduce here to our readership. An important goal of the editors-in-chief has always been to ensure that *Aging Cell* remains current and relevant to the scientific community at large. With a steady increase in the submission and publication of articles from many countries around the globe, we recognize the importance of maximizing the accessibility and impact of the research published in the journal. To maintain and promote our leading position, the publishers, with the support of the editorial team, and the Anatomical Society, have decided to convert *Aging Cell* to an author-pays open access model.

This change will not only comply with funder mandates, but more importantly, it will allow anyone and everyone around the world with internet access to read all material in *Aging Cell*, irrespective of field, host institution, location, or mode of internet access. Articles will be published under a Creative Commons license, and authors will be the copyright holders. All other editorial policies, such as those concerning the scope of the journal, the review process, and the length, number, and quality of accepted articles, will remain in place (visit our website, http://www.aging-cell.com/, for additional details).

We have also made a number of important changes to the *Aging Cell* website which we believe will enhance the experience of accessing content in the journal. The new website is more attractive visually and provides constantly updated information on features of interest in the journal as well as the aging field at large. To complement this, the content of *Aging Cell* and articles of interest in other journals can be followed on Twitter (https://twitter.com/AgingCell). Through an improved website and by keeping pace with social media, *Aging Cell* aims to develop into an online interaction hub for the aging research community.

An important strength of *Aging Cell* has always been its editorial team and policies, in particular the large and diverse editorial board. This board is comprised of internationally recognized leaders in all the subfields of biogerontology who act as supervising editors, managing the entire review process of assigned papers (including the choice of reviewers and rendering decisions). Hence, papers reviewed by *Aging Cell* receive personal attention from the top experts in the field. This rigorous process often necessitates extensive revisions and ensures that only the highest quality papers are published in the journal. Many authors have commented on the dramatic improvement of their papers during the review process. The membership of the editorial board is continuously updated, with several new members joining us in 2013 (visit our website, http://www.aging-cell.com/, for additional details).

We hope that all of you will share our excitement in these new developments. We would also like to take this opportunity to sincerely thank all the authors, reviewers, editorial board members, and managing editorial team for their hard work and dedication, and for making *Aging Cell* what it is: the flagship journal of the biology of aging. Above all, we would like to thank our readership – we hope you have found the content of the journal informative as well as important for your research. We welcome any comments or suggestions, and as always, we welcome your best papers.

